# Diagnosis and Management of Cirrhosis-Related Osteoporosis

**DOI:** 10.1155/2016/1423462

**Published:** 2016-10-20

**Authors:** Lívia Alves Amaral Santos, Fernando Gomes Romeiro

**Affiliations:** Department of Internal Medicine, Botucatu Medical School, Universidade Estadual Paulista (UNESP), Av. Prof. Mário Rubens Guimarães Montenegro, s/n, Distrito de Rubião Jr., 18 608 917 Botucatu, SP, Brazil

## Abstract

Management of cirrhosis complications has greatly improved, increasing survival and quality of life of the patients. Despite that, some of these complications are still overlooked and scarcely treated, particularly those that are not related to the liver. This is the case of osteoporosis, the only cirrhosis complication that is not solved after liver transplantation, because bone loss often increases after immunosuppressant therapy. In this review, the definitions of bone conditions in cirrhotic patients are analyzed, focusing on the more common ones and on those that have the largest impact on this population. Risk factors, physiopathology, diagnosis, screening strategies, and treatment of osteoporosis in cirrhotic patients are discussed, presenting the more striking data on this issue. Therapies used for particular conditions, such as primary biliary cirrhosis and liver transplantation, are also presented.

## 1. Introduction

In recent decades, advances in the management of cirrhosis complications and in liver transplantation have been increasing survival rates and improving the quality of life of cirrhotic patients. However, the longer survival of these patients has increased the risk of some extrahepatic manifestations such as osteoporosis. Regardless of the liver disease etiology, the presence of cirrhosis implies a risk of fractures two-fold higher than in noncirrhotic people [1]. Osteoporosis, the main bone disturbance among patients with liver insufficiency, is a systemic and progressive disease that affects bone mass and strength, thereby increasing the risk of fractures and compromising life quality due to pain and deformities [2]. Furthermore, this is the only cirrhosis complication that persists for years after liver transplantation [3–6].

Despite that, osteoporosis is often overlooked and few cirrhosis patients are submitted to exams to diagnose it. Even those who were diagnosed are sometimes precluded from starting a treatment due to the few options that can be offered. Consequently, many patients with liver cirrhosis also suffer from osteoporosis, which can have a big impact on them. In particular, patients receiving glucocorticoids and/or those submitted to liver transplantation suffer an additional decrease in their bone mass due to the use of immunosuppressant drugs. Therefore, some authors have advocated that bone densitometry must be part of the evaluation performed before orthotopic liver transplantation (OLT) [2, 7]. Furthermore, recent data have suggested that bone status must be assessed in all cirrhotic patients [8, 9].

The first studies of osteoporosis in liver diseases evaluated patients with alcoholic cirrhosis or chronic cholestatic diseases, such as primary biliary cholangitis (PBC) [10–15]. Then, other studies assessed patients before and after OLT [16, 17]. Most of them have shown that osteoporosis is common among all cirrhotic patients regardless of the liver disease etiology or the degree of liver impairment [7, 9, 18, 19]. Thus, the aim of this review was to evaluate the physiopathology, the impact, the diagnosis, and the management of osteoporosis in patients with liver cirrhosis, in order to show the more recent data and establish some comparisons between cirrhotic patients under different conditions.

## 2. Definition and Prevalence

As the population has been reaching older ages, the prevalence of primary and idiopathic osteoporosis has been increasing worldwide, with a global prevalence estimated at around 200 million [20]. According to the WHO definition, osteoporosis is diagnosed when bone density is less than 2.5 standard deviations below the peak value obtained from normal adults and adjusted for gender. It requires that the bone assessed be free from other systemic problems, including osteomalacia, or local abnormalities, such as osteophytes, extraskeletal calcifications, or deformities due to previous fractures [21].

A limitation of this definition is that the threshold was established from studies of postmenopausal Caucasian women, so there is not a single value that could be applied to all patients, such as those with liver diseases [1]. This may account for why many authors addressing bone impairment in patients with liver diseases have described it by employing the term “hepatic osteodystrophy.” However, this denomination also includes osteomalacia, which is caused by impaired bone mineralization and is not common among cirrhotic patients [2].

The many risk factors associated with bone loss include alcohol abuse, smoking, liver cirrhosis, neoplastic illness, malnutrition, prolonged glucocorticoid treatment (prednisone 5 mg/day for >3 months), kidney disease, vitamin D deficiency, and some hormonal disturbances such as diabetes, Cushing syndrome, hypogonadism, hyperparathyroidism, hyperthyroidism, and hypercalciuria [22, 23]. Prevalence in cirrhotic patients varies from 12 to 70% according to the diagnostic approach and the liver disease etiology [2, 8, 19, 23, 24]. The initial disease that caused the liver fibrosis is important in some particular conditions, such as cholestatic diseases, in which osteoporosis prevalence seems to be higher, varying from 20 to 44% even without an established diagnosis of cirrhosis and in proportion to the degree of liver insufficiency [1, 23, 25].

Patients with osteoporosis are susceptible to fractures of different bones such as vertebrae, femoral neck, and distal radio. The fractures occur when bones deform more than their peak strain or if they are not able to deform and exceed their peak stress [26]. Vertebral compressing fractures afflict 7% to 35% of cirrhotic patients, whereas the prevalence of peripheral fractures is around 10% [2, 27]. Again, the fracture rates are also higher in cholestatic diseases, varying from 13 to 22% according to the degree of liver insufficiency [1, 2, 4, 6, 25, 28, 29].

Although osteoporosis is asymptomatic in most cases, at five years after OLT it is related to symptoms associated with low quality of life scores because the patients report a decrease in bodily pain and physical function domains [5]. Low bone mineral density (BMD) before liver transplantation and the presence of a prior fracture are relevant predictors of bone loss after OLT. Once the patient is submitted to this procedure and starts taking immunosuppressive therapy, the loss of BMD is faster from the third to the sixth month after the surgery, and the incidence of fractures is between 6 and 65% [6]. Early bone loss after the surgery may affect the patients for years, making them susceptible to fractures even when BMD is being restored [4].

Remarkably, bone loss in liver cirrhosis is more severe among trabecular bones, such as vertebrae, with a lesser impact on the cortical ones. This pattern of increased vertebral damage is similar to some findings observed in the elderly, leading to compression fractures, disability, and spinal deformities [27, 32, 33]. Unexpectedly, most of these fractures are overlooked in noncirrhotic individuals [34]. A reasonable cause may be the fact that vertebral fractures can be less symptomatic than hip fractures, which occurs in individuals who are still able to walk [35]. On the other hand, hip fractures have a larger impact on mortality. Probably, this increase in mortality is due to the impact on patients' ability to walk and take care of themselves.

Prevention has been facilitated by BMD measurement, because it is the best predictor of fractures caused by osteoporosis. For each BMD reduction of one standard deviation, the risk of fractures is 2 to 3 times higher [21, 36]. Even so, BMD should be evaluated with additional information because there is not a single value to predict fractures in all cirrhotic patients [1].

## 3. Physiopathology

Although the mechanisms of cirrhosis-related osteoporosis are not fully understood, it is well known that the association between liver and bone diseases occurs due to an imbalance of bone turnover, which depends on osteoblastic and osteoclastic activity [22]. Most studies point to a more significant impairment in bone formation, suggesting that osteoporosis in cirrhotic patients is a multifactorial disease in which different mechanisms act together to reduce bone mass until achieving skeletal fragility [2]. Histological specimens from bones of cirrhotic patients with bone loss are similar to those obtained from elderly or postmenopausal women [37].

Part of the current knowledge is based on toxic effects and hormonal imbalances caused by liver insufficiency. In addition, chronic inflammation seems to play a role that is attributed to the small intestinal bacterial overgrowth caused by portal hypertension, leading to an increased flow of bacterial components into the portal vein [38]. The relevance of this process in hepatic decompensation was already evaluated by our group, showing that cirrhotic patients with hepatic encephalopathy remained hospitalized for more time when they presented higher levels of C reactive protein, a marker of systemic inflammation [39]. The inflammation process combined with immobilization is already recognized as a risk factor for bone loss. Thus, chronic inflammation in cirrhotic patients seems to be an additional cause that would account for the differences in regulators of bone remodeling and osteoclastogenesis observed in comparisons between liver disease patients with and without bone loss.

Prior studies found that serum tumor necrosis factor receptors and interleukin 6 (IL-6) levels were inversely correlated with BMD and that IL-6 can lead to bone loss by inducing the receptor activator of nuclear factor k*β* (RANKL) [40, 41]. RANKL is a member of the tumor necrosis factor family. It is a RANK ligand that is crucial for osteoclastic activation and differentiation. In a relevant study of noncirrhotic individuals from Italy, the authors showed that low serum RANKL was an independent predictor of nontraumatic fractures [42]. On the other hand, osteoclastogenesis is counteracted by osteoprotegerin (OPG), which is a RANKL receptor, making the balance between RANKL and OPG a chief point for regulating bone homeostasis. Results from patients with liver diseases showed an imbalance between RANKL and OPG, leading to bone loss [43–45].

Moschen et al. measured the soluble RANKL (sRANKL) in liver disease patients and found that it was higher than in controls, except in the cirrhotic subgroup. They also found higher OPG in these patients, in whom the highest levels were found in those with cirrhosis. Then, they showed that the OPG/sRANKL ratio was proportional to the degree of liver insufficiency and that it was higher in the subgroup of cirrhotic patients with osteoporosis and osteopenia. Of note, the sRANKL values were higher in patients with normal BMD in the lumbar spine. Furthermore, the authors found that RANKL+ cells were more prominent in liver biopsy specimens from liver disease patients. They hypothesized that the high sRANKL levels reflected increased bone turnover in liver disease patients and that the high OPG/sRANKL ratio might be an attempt to maintain bone homeostasis in these patients [45].

As the physiopathology of cirrhosis-related osteoporosis is not fully understood, other factors are briefly presented in this review in order to support a further discussion about possible treatment options.

### 3.1. Genetic Factors

Until now, there is not a single known genetic marker of osteoporosis predisposition. Some genetic polymorphisms are linked to the development of chronic cholestatic diseases, such as PBC. In this specific disease, insulin-like growth factor 1 (IGF-1) polymorphisms seem to have a higher influence on bone loss than the collagen type I*α*1 Sp1 polymorphism, which was previously evaluated in primary osteoporosis [46, 47]. Despite the reasonable knowledge on the possible role of these polymorphisms in PBC, more studies are still needed in this area [2, 48].

### 3.2. Hormonal Disturbances

Hypogonadism is a common finding in chronic liver disease. More than 90% of men with liver cirrhosis present low testosterone levels, which have an independent impact on mortality [49, 50]. There are many reasons for low levels of sexual hormones in these patients, including hypothalamic-pituitary-gonadal axis dysfunction [49]. Some symptoms found in hypogonadism are similar to those seen in advanced liver disease, hampering the ability to recognize which of them are the main cause of such patient complaints [50].

Hypogonadism is a cause of increased bone turnover, particularly in patients with hemochromatosis [27, 30–32, 51, 52]. Despite the iron overload effects, patients with hemochromatosis seem to be more prone to developing osteoporosis when they also have hypogonadism [30–32]. Some authors have hypothesized that low levels of sexual hormones (estrogen or testosterone) increase the osteoclasts life spam and decrease it in osteoblasts, leading to higher bone resorption than new bone synthesis [26, 30–32, 53]. Likewise, there are other hormones involved in liver diseases that can affect bone remodeling, such as osteocalcin.

Osteocalcin, a hormone secreted by osteoblasts, is involved in many steps of bone synthesis, such as calcium homeostasis, bone matrix mineralization, and osteoblastic proliferation [35]. Low osteocalcin levels have been found in patients with chronic liver diseases and are associated with bone loss [1, 30–32, 54, 55].

Another factor that can impair osteoblastic cells activity is a reduction in insulin-like growth factor 1 (IGF-1) levels. Since IGF-1 is produced in the liver, its deficiency is common in chronic liver diseases, impairing osteoblastic activity, collagen synthesis on bone matrix, and even bone mineralization [27, 54, 56, 57]. IGF-1 levels are lower in cirrhotic patients with osteoporosis when compared to those without osteoporosis, and they are also associated with the degree of hepatic insufficiency [57]. Of note, the IGF-1 replacement in animals with cholestatic disease is able to mitigate and partially reverse osteoporosis [1].

### 3.3. Toxic Effects

Alcohol is a well-known risk factor for osteoporosis in normal and cirrhotic populations [55]. Bone biopsies from patients addicted to alcohol who presented low levels of osteocalcin revealed decreased bone synthesis, but these levels were normalized after stopping alcohol consumption [30–32, 58, 59]. Moreover, these low osteocalcin levels can be added to malnutrition, hypogonadism, and other findings attributed to alcohol abuse.

Similar to some of these effects,* in vitro* studies showed a noxious effect on osteoblastic cells exposed to iron overload, another cause of decreased bone synthesis that accounts for osteoporosis in patients with hemochromatosis [27, 30–32, 60]. Thus, whereas hypogonadism has a dual effect on osteoclastic and osteoblastic cells, the toxic effects of alcohol and iron are more noticeable in relation to the bone synthesis. However, the role of bilirubin and biliary acids on bone loss seems to be even more complex.

Reduction of osteoblastic activity has been reported when unconjugated bilirubin levels are high [1, 55]. Nonclinical studies showed that bilirubin levels have an irreversible and dose-dependent impact on osteoblastic activity [22, 61, 62]. Another study showed that lithocholic acid can impair the effects of vitamin D on osteoblasts [63]. Some clinical studies found a progressive reduction in BMD, which was proportional to the degree of jaundice in patients with PBC or primary sclerosing cholangitis (PSC) [22, 64, 65]. However, another study did not find this correlation [66].

Glucocorticoids are often used after OLT and for patients with some liver diseases but are associated with bone loss [67, 68]. Of note, glucocorticoid-induced osteoporosis is a great concern in relation to the use of these drugs [69–71]. The bone side effects can be even worse when these drugs are associated with calcineurin inhibitors, impairing osteoblastic differentiation and increasing osteoclastic activity [8, 70, 72].

## 4. Other Factors

Vitamin D (25-hydroxyvitamin D) is a liposoluble substance that exerts important effects on bone metabolism. Low levels can be found in about one-third of liver disease patients, but severe deficiency is more common in those stricken by cirrhosis and/or cholestatic diseases, because jaundice can make them more prone to malnutrition, malabsorption, and suppressed skin synthesis [22, 73, 74]. Vitamin D deficits can cause bone loss due to increased bone turnover, thus increasing the risk of fractures [8, 75–77]. Calcium and vitamin D deficiency can lead to secondary hypoparathyroidism, which may increase bone turnover in cholestatic patients [2, 78]. Despite that, no clear association has been established between bone loss and calcium or bone loss and vitamin D levels [4, 22].

Vitamin K has an antiapoptotic effect on osteoblasts, and decreased levels can impair the synthesis of important proteins of bone matrix (osteocalcin and osteonectin) in patients with PBC [37, 79, 80]. It is also involved in the inhibition of osteoclastic differentiation [81–83].

Many studies have accessed the anorexigen effect of the adipokine leptin, which is also well known for its role in energy expenditure. It is mostly secreted by adipocytes and is involved in bone homeostasis, thus enhancing bone matrix synthesis, decreasing RANKL production, and increasing osteoblastic proliferation. Despite the decrease observed in cholestatic patients, not all hepatic diseases lead to suppressed leptin levels, and clear data on its role in osteoporosis are still lacking [1, 84].

Finally, smoking, lack of physical activity, malnutrition, and low body mass index are common findings among cirrhotic patients. As all of them are associated with osteoporosis both in cirrhotic and noncirrhotic patients, their avoidance is suggested in order to preserve bone health [85–88]. Even though most factors involved in cirrhosis-related osteoporosis are more closely linked to bone formation, malnutrition and alcohol abuse have a widespread effect because they can be involved in other risk factors mentioned above, such as leptin levels and vitamin deficiencies.

**Figure 1:**
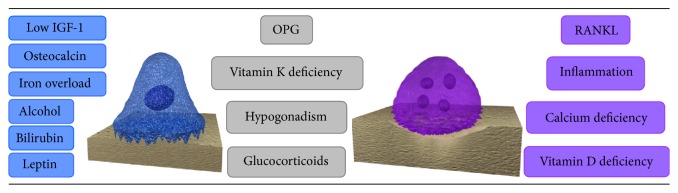
Factors that can be involved in cirrhosis-related osteoporosis by modulating the activity of osteoblastic and osteoclastic cells. The factors most related to osteoblastic activity are illustrated in the left column. The factors related to both osteoblastic and osteoclastic activity are displayed in the middle column. The factors most related to osteoclastic activity are shown in the right column. OPG = osteoprotegerin; RANKL = receptor activator of nuclear factor k*β*; IGF-1 = insulin-like growth factor 1. Although most factors are related to osteoblastic activity, malnutrition and alcohol abuse have a broad effect on bone loss because they can be involved in other risk factors displayed above, such as leptin levels and vitamin deficiencies.

Figure 1 presents some of these interactions observed in cirrhotic-related osteoporosis, focusing on its effects on osteoblastic and osteoclastic cells.

## 5. Diagnosis and Screening

Screening for osteoporosis is an important part of cirrhosis management, but it is not always performed [89]. Moreover, densitometry indications have been applied just for patients considered for OLT and for those with cholestatic diseases or those under glucocorticoid therapy.

It has been accepted that bone mass is the best measurement for evaluating skeletal strength [90]. Thus, the guidelines indicated that all cirrhotic patients should be screened by an initial dual-energy X-ray absorptiometry (DXA) exam, emphasizing that a normal result should never be sufficient to discard the risk of osteoporosis and that any additional risk factor must lead to a higher level of awareness [90]. The exam allows measurement of BMD and should be repeated after 2 to 3 years to assess significant bone loss, particularly in the presence of the aforementioned risk factors [48, 90]. For cholestatic patients with more than one risk factor and for those who recently started glucocorticoid therapy, DXA should be repeated in one year [48]. In addition, BMD should be measured again before OLT [91].

Regarding DXA accuracy, some limitations must be taken into account. The presence of ascites causes underestimation of the real BMD value. This problem is even worse in the lumbar spine and in patients with a large volume of ascites, leading to vertebral BMD values of 4.2 to 7% higher after paracentesis and changing the diagnosis of 12% of patients [91, 92]. Therefore, it is recommended to measure BMD just after paracentesis to not overestimate bone alterations [91, 92].

A lateral vertebral X-ray can be important as a complimentary exam to search for dorsal and lumbar spine fractures [2]. In cases with local deformities, the corresponding BMD values can be altered and are not reliable. Subtle deformities from previous fractures can cause significant changes in BMD, leading to misinterpretation of the values obtained. To avoid this inaccuracy, clinicians must ask about previous traumas and look for them during the physical exam. Whether the risk of local deformities exists, a two-dimensional X-ray exam can be used to check the vertebrae and the femoral neck before ordering a more expensive exam. Any surface alteration on the cortical layer or signals of lumbar vertebrae collapse are reasons to change the area used for BMD measurements.

Some lab tests can be also useful for evaluating bone metabolism, including serum calcium, vitamin D, phosphorus, osteocalcin, procollagen I carboxyterminal peptide, and parathyroid hormone (PTH), as well as urinary amino telopeptides of collagen I and urinary calcium [2, 93].

In a recent trial, body mass index, leukocyte count, serum bilirubin, and transient elastography values were independently associated with low BMD [19]. In another study developed by our group, we found that high PTH levels and low handgrip strength could be used as accurate predictors of low BMD in the lumbar spine of cirrhotic patients, showing values of these variables that can be used as cutoff points to indicate which patients should be submitted to DXA [9].

Since there are noninvasive measures that can be used as surrogate markers of osteoporosis, bone biopsies are rarely used in cirrhotic patients. In cases of bone loss, bone biopsies from cirrhotic patients have findings that are quite similar to those seen in elderly people [37]. The exception is alcoholic liver cirrhosis, in which impaired osteoblastic activity leads to increased resorption surfaces, lowering the trabecular bone volume [13, 94].

## 6. Treatment

Most recommendations for osteoporosis treatment in cirrhotic patients were based on results obtained from trials assessing postmenopausal women and smaller studies including patients with dissimilar liver diseases [54]. As a rule, the modifiable risk factors should be taken into account in order to minimize the bone loss, starting from lifestyle changes such as tobacco and alcohol cessation and increasing physical activity as much as possible to improve spinal biomechanics [27]. Furthermore, a balanced diet must be prescribed because nutritional deficits are common among cirrhotic patients.

It is well known that patients with osteoporosis and/or fractures associated with skeletal fragility must be treated, and part of the treatment has been also recommended for patients with osteopenia when they present additional risk factors for bone loss, such as those with cholestatic diseases [2].

For the majority of patients, osteopenia (*T* scores between 1 and 2.5 standard deviation from normal values) is not considered a disease. Even so, it leads to awareness that bone loss is already present. For instance, Guañabens et al. reported that patients with PBC whose* T* scores are below 1.5 standard deviation from normal values had a significant risk for vertebral fractures, showing that patients without osteoporosis also suffer fractures and should be considered for receiving prophylactic therapy [25].

### 6.1. Calcium and Vitamin D Supplementation

Although the ideal amount of calcium ingestion has been debated for decades, calcium supplementation is still part of osteoporosis treatment. The total calcium intake should achieve a daily ingestion of 1.0 to 1.5 grams according to age and other factors. Preferably, calcium from diet should be chosen, because it would facilitate the patients' compliance. Moreover, data on cardiovascular risk in patients taking calcium supplements are still unclear [95, 96]. Nevertheless, as this risk was not evaluated in specific populations, it should not preclude the use of these supplements [97]. The supplement most widely consumed by patients is calcium carbonate, which must be ingested along with foods to increase absorption. Calcium citrate is more suitable for patients with achlorhydria or other conditions that could impair gastrointestinal absorption. Another important recommendation is that calcium tablets should never be ingested together with fluoroquinolones, tetracycline, bisphosphonates, phenythoin, or levothyroxine, because the supplements impair the absorption of these drugs.

Oral 25-hydroxyvitamin D supplementation can be prescribed at a dose of 260 *μ*g every 2 weeks. Since calcitriol (1,25-dihydroxycholecalciferol) is the final active metabolite of vitamin D, it seems to be a better treatment to these patients. Calcitriol is usually prescribed as a daily oral dose of 800 U but can also be taken at a weekly dose of 5000 U [2]. In a clinical trial in which calcitriol (0.5 mg twice per day) was given to 38 cirrhotic patients for 12 months, the authors showed that the supplementation was the only factor significantly related to BMD increasing [98]. Although calcium and vitamin D are widely used for osteoporotic patients, evidence confirming that these supplements could reverse or avoid osteoporosis is unclear [27].

In a systematic review of calcium and vitamin supplementation to prevent or treat osteoporosis in the general population, Bolland et al. found small benefits in fracture avoidance from calcium and vitamin D supplements. The authors reported that calcium supplements have some effect on reducing vertebral fractures; however, the number needed to treat (NNT) in order to prevent one vertebral fracture was 489 patients taking the supplements for 6.2 years [99]. The results on isolated vitamin D supplementation were even weaker, suggesting no benefit of adding vitamin D to calcium supplements. A small effect on hip-fracture risk was produced by both supplements [99].

In addition, data from elderly noncirrhotic patients showed that adherence to these supplements decreases through time. Less than half of them are still taking it after one year if educational interventions are not provided [100]. Data from cirrhotic patients are not so common, but some studies of patients with PBC showed that calcium and vitamin D were not able to change their BMD when compared to hormonal replacement therapy [101, 102]. Even so, it is always worthwhile to test for vitamin D deficiency in cholestatic patients, particularly those taking cholestyramine, which impairs its absorption [27].

### 6.2. Hormonal Replacement

Hormonal replacement can be a valuable approach for patients with hypogonadism, increasing BMD values in both genders and decreasing the risk of fractures in women [102–105]. In contrast, one of the studies of PBC patients suggested that those receiving estrogens could present a high risk of cholestasis [106]. Moreover, the risk of developing hepatocellular carcinoma in patients receiving testosterone is another concern, although there are no clinical data to confirm this hypothesis [21, 107].

Isoniemi et al. treated 33 postmenopausal women for two years after OLT. Given some reasonable concern about the procoagulant effect of oral hormonal replacement therapy, the authors chose a transdermal estradiol treatment, which was given in the first six months after OLT. After one year of treatment, the authors documented respective lumbar and femoral BMD increases of 5.3% and 3.3%. Of note, the increases were not as great after the second year (1.2% at both sites). Furthermore, they still documented a marked improve in the patients' lipid profile [108]. Other studies also reported similar BMD results from transdermal hormonal replacement in patients with PBC, showing that this type of treatment is safe and can bring other benefits to patients [101, 102, 109, 110].

### 6.3. Calcitonin

Calcitonin is able to inhibit osteoclastic activity, but the use of this hormone for cirrhotic patients is still controversial. In the former study, women stricken by PBC and other liver diseases had bone density measured at two moments before the treatment, showing a significant decrease in PBC cases in a six-month period. Then, 13 patients who had reduced bone density received a four-month treatment composed of calcitonin (40 U) thrice a week together with daily calcium and vitamin D supplements, which curbed their bone loss in comparison to the nontreated patients [111]. The results were confirmed by a further study at the same university, when the improvement was observed only in patients who had suffered a more pronounced bone loss and received the treatment. In this trial, calcitonin was administered on a schedule similar to the previous one, but for 3 years [112]. In contrast, another study reported that calcitonin given for 6 months was ineffective at increasing BMD in PBC patients [113].

In a study performed after OLT, 17 patients received a daily 40 IU dosage of intramuscular calcitonin for 15 days every 3 months combined with daily calcium supplement (1 g). After one year, the improvement in vertebral BMD was comparable to that observed in 23 patients receiving 400 mg of sodium etidronate given 15 days every 3 months [93]. Taken together, these results suggest that the beneficial effect of calcitonin in patients with liver diseases is more pronounced in those who present a faster bone loss.

### 6.4. Sodium Fluoride and Raloxifene

Sodium fluoride is well known for increasing lumbar spine bone mass in osteoporotic patients [114]. In a small randomized controlled trial, it was given to 7 PBC patients at a daily dosage of 50 mg with calcium and vitamin D supplements, while the placebo group (*n* = 8) received only the supplements. The results indicated that sodium fluoride halted the bone loss compared to the placebo [115]. The same group completed another trial comparing sodium fluoride with etidronate in two groups of 13 women with PBC. After two years of treatment, the authors found a subtle increase in vertebral BMD only in the fluoride group. Despite the occurrence of two vertebral fractures in this group, etidronate was considered a safe treatment [116]. More studies are needed to confirm efficacy and safety of sodium fluoride for cirrhotic patients.

Raloxifene is a second-generation selective estrogen-receptor modulator that shows estrogenic actions on bones. It has been used to treat osteoporosis in patients without liver diseases, but not yet in cirrhotic patients. A prior study was performed in nine postmenopausal women with PBC, suggesting a possible benefit in lumbar spine BMD [117]. The lack of studies in patients with liver cirrhosis hampers recommending it for this population.

### 6.5. Bisphosphonates

Anticatabolic drugs seem to be a good option for treating osteoporosis in cirrhotic patients, because they are stricken by several metabolic alterations. Bisphosphonates appear to be helpful in the treatment of cirrhosis-related osteoporosis, because these drugs attach to the bone surface and prevent resorption (the so-called “antiresorptive” effect) [118]. Bisphosphonates are able to increase bone mass in postmenopausal women, but concerns about the potential risk of ulceration on esophageal varices have reduced the number of studies in cirrhotic patients, hampering the extrapolation of any data to them. After some trials showing interesting results, this risk has been considered lower than formerly estimated [21].

One of the first trials that showed a significant effect of bisphosphonates on bone mass was conducted in 1998. Ninety patients received pamidronate 60 mg every 3 months before OLT and for 9 months after the procedure. Before treatment, seven subjects had vertebral fractures (most of them had PBC and lower BMD values compared to other patients). Jaundiced patients also received calcium and vitamin D. Since this routine treatment was adopted, symptomatic fractures were no longer registered, leading the authors to recommend it [119].

As commented above on sodium fluoride, results obtained from etidronate showed that it was only able to prevent bone loss, which did not change in two years of treatment. This finding prompted the authors to consider that etidronate was not capable of increasing bone mass in PBC patients [116, 120]. Thus, they performed a 2-year randomized trial using alendronate 10 mg daily for 13 PBC patients, achieving good BMD results and no significant side effects [120]. Similar findings were also observed in a trial using 70 mg weekly throughout one year [121]. Another trial comparing monthly ibandronate versus weekly alendronate (150 mg and 70 mg, resp.) for PBC patients found comparable effects on BMD but a higher compliance with the monthly treatment [122]. Yet, it is important to point out that these trials included only patients with PBC.

Other studies assessed the efficacy and safety profile of parenteral drugs, such as pamidronate. Data obtained from trials using this drug in cirrhotic patients are also limited to few studies. Ninkovic et al. assessed 99 patients randomized to receive pamidronate 60 mg intravenously in a single-dose versus no treatment before OLT. The authors did not find any difference in fracture rates or BMD between the former and latter groups [123]. Twelve of these patients were also included in another study assessing iliac-crest biopsies before and three months after OLT, showing a lesser degree of bone resorption in patients who received pamidronate (*n* = 7) [124].

Dodidou et al. evaluated 21 OLT patients who had taken infusions of pamidronate (30 mg) every three months after the surgery, combined with vitamin D and calcium supplements. The authors also assessed 13 cardiac transplant patients taking the same regimen and compared data from transplanted subjects with a historical reference group. They found a significant increase in lumbar spine and femoral neck BMD in those who received pamidronate, which persisted during the second year of treatment [125]. In another trial, this same treatment was administered to 43 patients after OLT, comparing BMD with 38 controls. Twenty-four patients (54% with osteoporosis) and all the controls (23% with osteoporosis) presented a significant increase in lumbar spine but not in femoral neck BMD [126].

Millonig et al. investigated the role of alendronate 70 mg weekly combined with calcium and vitamin D supplements for preventing bone loss in 98 patients who had osteoporosis or osteopenia and started receiving this drug after OLT. The authors assessed BMD before OLT and every year after until 48 months to document bone changes in this interval. A significant improvement was observed between 4 and 12 months after OLT in lumbar spine BMD of osteoporotic patients, with subtle changes in the subsequent years. In femoral neck, osteoporotic patients increased their BMD between 4 and 12 months and until 3 years after OLT. Only two patients discontinued the drug because they reported abdominal discomfort, and the drug was changed to pamidronate 30 mg monthly [127].

Crawford et al. evaluated the effect of zoledronic acid 4 mg on BMD of 32 patients submitted to OLT (18% of whom had osteoporosis). The drug infusion was given within seven days after OLT and repeated at one, three, six, and nine months after the procedure. Calcium and vitamin D were also administered. The authors compared the results with those of 30 patients receiving placebo (10% with osteoporosis) and reported that lumbar spine, femoral neck, and total hip BMD measurements favored zoledronic acid at the first 3 and 6 months after OLT. Fifteen patients did not complete the study, four of them because of fractures (2 nonvertebral fractures in the treatment group and 2 vertebral fractures in the placebo group). Despite the benefits in BMD, the drug induced hyperparathyroidism and postinfusion hypocalcemia [128].

Atamaz et al. conducted a trial with 44 subjects receiving alendronate 70 mg weekly and 40 patients in a control group. Initially, 98 patients were recruited after OLT, but three of them died, one discontinued the treatment because noncompliance, and two subjects in the alendronate group had gastrointestinal distress. The drug was given for one month and led to a significant improvement in mean BMD at lumbar spine, femoral neck, and total femur (5.1 ± 3.9%, 4.3 ± 3.8%, and 3.6 ± 3.8%, resp.). Musculoskeletal pain and upper gastrointestinal adverse events were present in 38.6% and 29.5% of subjects, respectively. There were two nonvertebral fractures in the control group and none in the alendronate group. Seven patients in the control group and three in the alendronate group had new vertebral fractures. It was noteworthy that most of the vertebral fractures were detected only in X-ray exams [129].

Bodingbauer et al. recruited 96 patients after OLT to receive 4 mg of zoledronic acid monthly plus calcium and vitamin D or only calcium and vitamin D. The treatments were administered for one year after OLT. Thirty-five patients in the study group and 34 in the control group were monitored for 2 years. Pyrexia and musculoskeletal pain were more common in the study group. The authors reported a significant (*p* = 0.05) reduction of vertebral fractures in the study group (four subjects) compared to the control group (11 subjects). Six months after OLT there were no significant differences on lumbar spine BMD between the groups, but a small difference in femoral neck BMD [130]. Part of the study sample was reevaluated to reassess data from transiliac biopsies, showing that zoledronic acid reduced bone turnover in 21 patients [131].

Monegal et al. performed a randomized controlled trial comparing pamidronate versus placebo in 10 Spanish centers. They analyzed 32 patients in the experimental group and 34 in the placebo group. Pamidronate 90 mg was given within 7 to 12 days after OLT and again three months after the surgery. All patients received calcium and vitamin D supplements. Seven subjects in the experimental group and three in the placebo group had fractures within the first year after OLT, most of which were in vertebrae. The number of adverse events did not differ between the groups. The authors reported a significant improvement in lumbar BMD achieved by pamidronate use (2.9% in the treatment group and 1% in the control group). However, both groups displayed a subtle decrease in femoral neck BMD. Additionally, trochanteric BMD was reduced only in the placebo group [132].

These prior trials evaluated specific populations, such as PBC patients or those submitted to OLT, but none of them had included only patients with cirrhosis during the treatment. Given the toxic effect of cholestasis and immunosuppressant drugs on bone health, it is somewhat difficult to extrapolate the data from these studies to all cirrhotic patients. Therefore, it has provoked several discussions about the possibility of performing similar studies of cirrhotic individuals.

Then, Yurci et al. studied a different sample composed of patients with cirrhosis (31 subjects) and viral hepatitis (50 subjects) who had reduced* T* scores in at least one region. The authors divided their sample into six groups according to the drugs administered (salmon calcitonin 200 IU daily, calcium, vitamin D, and different dosages of alendronate). Most cirrhotic patients had compensated liver disease (18 subjects out of 31). Six patients with decompensated cirrhosis died during the study, but none of them was receiving bisphosphonates. Seven cirrhotic patients had the treatment discontinued. The authors concluded that alendronate prevented trabecular and cortical bone loss in their sample, with no significant side effects [118]. Unfortunately, the groups had few subjects with cirrhosis.

In a recent trial performed by Bansal et al., 215 cirrhotic patients were recruited to participate, of whom 47 had osteoporosis and received a monthly ibandronate dosage of 150 mg combined with calcium and vitamin D supplements for six months. The major cause of cirrhosis was alcoholic liver disease, and most patients had decompensated cirrhosis, esophageal varices, and ascites. This last finding was present in 175 subjects, bringing concern about the precision of BMD measures, as previously mentioned in this review and by other studies. Four patients with osteoporosis had fractures, 16 died, and 12 lost follow-up, so that only 19 completed the study. These 19 patients had a significant improvement in mean BMD (from 0.81 ± 0.07 to 0.88 ± 0.07) and* T* scores (from −3.28 ± 0.72 to 2.45 ± 0.45) [19].

## 7. Conclusions

As cirrhotic patients have been submitted to many treatments to achieve better survival, cirrhosis-related osteoporosis has become more common, especially among patients who present any other risk factor for bone loss, such as cholestatic diseases, alcohol abuse, hypogonadism, or the other factors previously mentioned in this review. Furthermore, osteoporosis is the only cirrhosis complication that worsens after liver transplantation. Thus, health professionals must become aware of this condition in order to diagnose it as soon as possible. Many screening strategies can be helpful for showing which patients should be submitted to specific exams, thereby reducing the budget of a whole population screening.

Once osteoporosis is diagnosed in cirrhotic patients or in those who were already submitted to OLT, it is important to control the risk factors that can increase bone loss, such as alcohol consumption, smoking, sedentary lifestyle, and glucocorticoid use. Dietary calcium intake must be checked, and calcium obtained from foods is generally preferable to tablets, which can impair the absorption of some drugs. Vitamin D levels should be measured while supplements seem to be valuable in specific conditions, such as cholestatic diseases. Since calcium and vitamin D supplementation seem insufficient to curb bone loss in this population, additional therapy must be prescribed.

Most trials that assess osteoporosis treatment in cirrhotic subjects had a limited sample and a short follow-up. Studies on hormonal replacement therapy for patients with hypogonadism achieved interesting results in women, but concerns about hepatocellular carcinoma prevented similar trials in men. Even among women with PBC or after OLT, the results were more striking in the first year of estrogen therapy. Despite the exciting data on efficacy and safety reported, more studies are still required to evaluate the beneficial effects of hormonal replacement on the bone health of cirrhotic patients. The same can be said about calcitonin, sodium fluoride, and raloxifene.

Notwithstanding the same problem regarding small samples, clinical trials with bisphosphonates combined with calcium and vitamin D supplements have achieved encouraging results. However, concerns about the risk of mucosal damage on esophageal varices have reduced the number of studies with cirrhotic patients, and most trials included only subjects who were submitted to OLT under different immunosuppressant regimens, hampering the ability to reach conclusions that could be applied to patients who still have liver cirrhosis. For now, the available results suggest that some bisphosphonates are safe and can really improve the bone health of patients with cirrhosis. It is important to know that some side effects can prevent the use of these drugs for all patients and that most data were obtained from surrogate markers and not from fracture incidence. Therefore, more studies are also needed to clarify the best options for this population [133].
